# Daikenchuto and Processed Ginger Alleviate Liver Ischemia–Reperfusion Injury by Enhancing Anti‐Inflammatory Effects and HO‐1 Production

**DOI:** 10.1002/jgh3.70478

**Published:** 2026-10-11

**Authors:** Kenichi Saga, Yoichiro Uchida, Hiroshi Kawamoto, Hirofumi Hirao, Yosuke Umino, Masaaki Ito, Yuki Kidoguchi, Junya Kawasoe, Kosuke Tanaka, Kentaro Kadono, Shoichi Kageyama, Kojiro Taura, Hiroaki Terajima, Takeshi Watanabe, Etsuro Hatano

**Affiliations:** ^1^ Department of Surgery, Graduate School of Medicine Kyoto University Kyoto Japan; ^2^ Department of Gastroenterological Surgery and Oncology Medical Research Institute KITANO HOSPITAL Osaka Japan; ^3^ Division of Immunology, Institute for Frontier Life and Medical Sciences Kyoto University Kyoto Japan

**Keywords:** 6‐shogaol, daikenchuto, heme oxygenase‐1, liver ischemia–reperfusion injury, processed ginger

## Abstract

**Background and Aim:**

Daikenchuto (DKT), a Kampo medicine, is widely used in surgical practice because of its pro‐motility effects on the gastrointestinal tract. Although its anti‐inflammatory effects in the gastrointestinal tract have been well documented, its role in hepatic ischemia–reperfusion injury (IRI) remains unclear. This study investigated the protective effects of DKT and its individual components against hepatic IRI.

**Methods:**

Male C57BL/6 mice were fed a diet containing 3.6% DKT or its individual herbal components for 1 week before 60 min of partial hepatic ischemia. Liver injury was evaluated based on serum liver enzyme levels, histology, and TUNEL staining. Portal venous adrenomedullin (ADM) levels and hepatic heme oxygenase‐1 (HO‐1) expression were also assessed. In vitro, peritoneal macrophages were stimulated with High Mobility Group Box 1 (HMGB1) to examine the effects of DKT and 6‐shogaol (6SG).

**Results:**

DKT significantly reduced serum alanine aminotransferase levels, necrotic area, and hepatocyte apoptosis compared to the control. Although single‐herb doses equivalent to those in the DKT formula did not confer protection, a threefold dose of processed ginger reproduced the hepatoprotective effects of DKT. DKT did not increase portal ADM levels. In contrast, both DKT and 6SG significantly upregulated HO‐1 expression in vivo and suppressed the production of inflammatory cytokines in HMGB1‐stimulated macrophages in vitro.

**Conclusion:**

DKT attenuates liver IRI via HO‐1 induction and suppression of macrophage‐mediated inflammation, rather than via ADM elevation. These protective effects may be mediated, at least in part, by processed ginger.

**Abbreviations**
6SG: 6‐shogaol
ADM: adrenomedullin
AU: arbitrary units
CD: control diet
CGRP: calcitonin gene‐related peptide
CXCL: C‐X‐C motif chemokine ligand
DAMPs: damage‐associated molecular patterns
DKT: daikenchuto
ELISA: enzyme‐linked immunosorbent assay
ERAS: enhanced recovery after surgery
HMGB1: High Mobility Group Box 1
HO‐1: heme oxygenase‐1
IL: interleukin
IR: ischemia and reperfusion
IRI: ischemia and reperfusion injury
JZP: Japanese zanthoxylum peel
KCNK9: potassium two pore domain channel subfamily K member 9
PBS: phosphate‐buffered saline
PG: processed ginger
POI: postoperative ileus
qRT‐PCR: quantitative real‐time polymerase chain reaction
RIA: radioimmunoassay
sALT: serum alanine aminotransferase
SD: standard deviation
TNF‐α: tumor necrosis factor‐α
TRP: transient receptor potential
TUNEL: TdT‐mediated dNTP nick end labeling
WT: wild‐type
ZnPP: zinc protoporphyrin IX

## 1 Introduction

Hepatic ischemia–reperfusion injury (IRI) is an unavoidable complication associated with liver resection and living donor liver transplantation and is a major contributor to postoperative liver dysfunction and/or failure [1, 2]. Inflammatory responses play a pivotal role in the development of hepatic IRI, where damage‐associated molecular patterns (DAMPs) released from necrotic cells, particularly high mobility group box 1 (HMGB1), activate immune cells such as macrophages, thereby initiating and exacerbating inflammation [3, 4, 5, 6]. Although hepatic IRI has clinical significance, effective therapeutic strategies remain limited, and novel interventions are urgently required.

Postoperative ileus (POI) is another common complication following open abdominal surgeries, such as liver resection and transplantation, which hinders postoperative recovery [7]. Daikenchuto (DKT) is a traditional Japanese medicine approved as an ethical Kampo formulation by the Ministry of Health, Labour and Welfare of Japan. It is composed of processed ginger, japanese zanthoxylum peel, ginseng, and maltose, and has been reported to enhance gastrointestinal motility [8] and improve intestinal blood flow [9]. DKT has shown promise in alleviating abdominal symptoms associated with POI [10] and may also contribute to improved nutritional status in the postoperative period [11], making it a focus of interest within the framework of enhanced recovery after surgery (ERAS) protocols.

The mechanisms underlying the pro‐motility effects of DKT are thought to involve the promotion of acetylcholine release from intrinsic cholinergic nerve terminals via 5‐HT_3_ and 5‐HT_4_ receptors [12, 13], as well as through the inhibition of the potassium channel subfamily K member 9 (KCNK9) channel [14]. Additionally, improvement in intestinal blood flow is associated with increased adrenomedullin (ADM) production induced by DKT and its ginger‐derived constituent 6‐shogaol (6SG) [15]. The anti‐inflammatory effects of DKT have also been reported in experimental models of enteritis [16, 17]. DKT has recently been applied in hepatobiliary surgery, and clinical studies have suggested its potential benefits [7]. Experimental research has shown that DKT may improve portal blood flow and exert favorable effects on the liver [18]. However, whether DKT exerts protective effects against hepatic IRI remains to be elucidated. Therefore, this study aimed to investigate whether DKT has protective effects against hepatic IRI using an experimental model.

## 2 Materials and Methods

### 2.1 Animals

Male C57BL/6 mice (8–10 weeks old, weighing 20–25 g) were purchased from Shimizu Laboratory Supplies (Kyoto, Japan). All animals were maintained under specific pathogen‐free conditions and received humane care in accordance with the Guide for the Care and Use of Laboratory Animals. All experimental protocols were approved by the Animal Research Committee of Medical Research Institute KITANO HOSPITAL, Osaka, Japan.

### 2.2 Mouse Diets

The control ordinary diet (CD) was an AIN‐93G diet. DKT was generously provided by Tsumura & Co. (Tokyo, Japan). DKT used in this study consisted of herbal medicinal extract powder and maltose powder at a ratio of 1.25:10 (w/w). The herbal medicinal extract powder was manufactured by Tsumura & Co. (Tokyo, Japan) as an intermediate product of TSUMURA Daikenchuto Extract Granules for Ethical Use (extract powder without excipients; Lot No. 2180100010).

The extract quality was standardized based on the Good Manufacturing Practice and in accordance with the *Japanese Pharmacopeia* (JP). Details of the manufacturing process are available on the company website (https://www.tsumura.co.jp/english/kampo/). Briefly, the extract powder was prepared by spray‐drying a hot‐water extract mixture of the following three crude drugs in the proportions indicated: Japanese Zanthoxylum Peel (ZANTHOXYLI PIPERITI PERICARPIUM) [Zanthoxylum piperitum De Candolle (Rutaceae), pericarpium] (2.0), Processed Ginger (ZINGIBERIS RHIZOMA PROCESSUM) [Zingiber officinale Roscoe (Zingiberaceae), rhizome] (5.0), and Ginseng (GINSENG RADIX) [Panax ginseng C. A. Meyer (Panax schinseng Nees) (Araliaceae), radix] (3.0).

The crude drugs were identified based on their external morphology and marker constituents, according to the JP and company standards.

The experimental diet containing 3.6% DKT mixed with an AIN‐93G diet was manufactured by Oriental Yeast Co. (Tokyo, Japan). To evaluate the effects of the individual crude drugs contained in DKT, experimental diets containing each crude drug were prepared based on the composition of DKT (0.2% processed ginger, 0.12% ginseng, and 0.08% Japanese zanthoxylum peel). Diets containing three‐fold concentrations of each crude drug were prepared. The CD was prepared from AIN‐93G using the same manufacturing process as the experimental diets.

### 2.3 Liver IRI Model

A well‐established mouse model of partial warm liver IRI was used [19, 20, 21]. The mice were anesthetized with isoflurane and administered heparin (100 U/kg) via an intravenous injection to prevent coagulation. An atraumatic vascular clip was applied to occlude the arterial, portal venous, and bile duct supplies to the left and middle lobes of the liver. Ischemia was maintained for 60 min at room temperature, after which the clip was removed to initiate the reperfusion. After 6 h of reperfusion, the mice were sacrificed, and liver samples were collected for analysis. Tissues were either fixed in 10% formaldehyde overnight for histological analysis or flash‐frozen in liquid nitrogen for molecular and biochemical analyses.

Sham‐operated control mice underwent identical procedures without vascular occlusion and served as the baseline controls.

### 2.4 Hepatocyte Function

Serum alanine aminotransferase (sALT) levels, which are used to measure liver injury, were determined using a standard spectrophotometric method with an automated clinical analyzer (JCABM9030, JEOL Ltd., Tokyo, Japan).

### 2.5 Histology

Liver injury was assessed on H&E‐stained paraffin sections using the modified Suzuki's criteria (0–4 scale for congestion, vacuolization, and necrosis) [22] and evaluated blindly in ten fields per sample.

### 2.6 Apoptosis Assay

Apoptosis in 5‐μm‐thick liver paraffin sections was detected using the terminal deoxynucleotidyl transferase‐mediated deoxyuridine triphosphate nickend labeling (TUNEL) method with an in situ Apoptosis Detection Kit (Takara Bio, Kyoto, Japan), according to the manufacturer's instructions.

### 2.7 Quantitative Reverse‐Transcription Polymerase Chain Reaction (qRT‐PCR)

Total RNA was extracted from the liver tissues using an RNeasy Mini Kit (QIAGEN, Venlo, the Netherlands). Complementary DNA was prepared using the PrimeScript RT Reagent Kit (TAKARA BIO, Kusatsu, Japan). qRT‐PCR was performed using a StepOnePlusTM Real‐Time PCR System (Life Technologies, Tokyo, Japan). The primers used to amplify specific gene fragments are listed in Table 1:. Target gene expression was calculated as the ratio of the target gene to the housekeeping gene β‐actin.

**TABLE 1:** The qRT‐PCR primer sequences.

Primer name	Primer sequences (5′–3′)
TNF‐α‐Forward	AGGGTCTGGGCCATAGAACT
TNF‐α‐Reverse	CCACCACGCTCTTCTGTCTAC
IL‐6‐Forward	ACCAGAGGAAATTTTCAATAGGC
IL‐6‐Reverse	TGATGCACTTGCAGAAAACA
IL‐1β‐Forward	CAGGTCGCTCAGGGTCACA
IL‐1β‐Reverse	CAGAGGCAAGGAGGAAACACA
CXCL1‐Forward	GCCTATCGCCAATGAGCTG
CXCL1‐Reverse	ATTCTTGAGTGTGGCTATGA
CXCL2‐Forward	CAGACAGAAGTCATAGCCAC
CXCL2‐Reverse	TTCCAGGTCAGTTAGCCTTG
HO‐1‐Forward	CAAGCCGAGAATGCTGAGTTCATG
HO‐1‐Reverse	GCAAGGGATGATTTCCTGCCAG
β‐Actin‐Forward	AGAGGGAAATCGTGCGTGAC
β‐Actin‐Reverse	CAATAGTGATGACCTGGCCGT

### 2.8 The Concentration of HMGB1 in the Inferior Vena Cava

Serum HMGB1 levels were quantified using an HMGB1 ELISA Kit Exp (Shino‐Test).

### 2.9 The Concentration of ADM in the Portal Vein Blood

Serum ADM concentrations were determined using a commercially available Radioimmunoassay (RIA) Kit (Phoenix Europe GmbH, Karlsruhe, Germany). Serum samples (1 mL) from the portal vein were diluted 1:1 with 0.1% alkali‐treated casein in phosphate‐buffered saline (PBS; pH 7.4) and applied to pre‐washed Sep‐Pak C18 cartridges (Waters Corporation, Milford, MA, USA) to remove ADM‐binding proteins such as complement factor H. The peptide‐containing fraction was eluted with 80% isopropanol containing 0.125 N HCl and lyophilized overnight. ADM levels in the dried extracts were quantified according to the manufacturer's RIA protocol.

### 2.10 Immunofluorescence

Frozen mouse liver samples (5 μm) were stained with rabbit anti‐heme oxygenase (HO‐1) antibody (Enzo Life Sciences, Farmingdale, NY, USA). Signals were visualized using a secondary Alexa Fluor antibody.

### 2.11 Reagents

Zinc protoporphyrin IX (ZnPP), an HO‐1 inhibitor, was purchased from FUJIFILM Wako Pure Chemical Corporation (Osaka, Japan). ZnPP was dissolved in 0.2 N NaOH, adjusted to pH 7.4 with 1 N HCl, diluted with saline, and administered to mice intraperitoneally at a dose of 25 mg/kg body weight 1 h before the ischemic insult.

### 2.12 Cell Cultures

Thioglycollate‐elicited peritoneal macrophages were isolated as previously described [23] and seeded into 24‐well culture plates at a density of 2.0 × 10^5^ cells/well in 0.5 mL of medium. The cells were incubated for 3 h at 37°C in a humidified 5% CO_2_ atmosphere. After incubation, non‐adherent cells were removed by washing three times with PBS. Adherent macrophages were treated with various concentrations of DKT and 6SG (*n* =  5 per group) for 30 min, followed by stimulation with bovine HMGB1 (1 μg/mL; Chondrex, Redmond, WA, USA). After 48 h, culture supernatants were collected and stored at −80°C for subsequent TNF‐α measurement using ELISA. The adherent cells were lysed in a buffer containing protease and phosphatase inhibitors, and the lysates were stored at −80°C until western blot analysis.

### 2.13 Statistical Analyses

All statistical analyses were performed using Prism 9 software (GraphPad Software Inc., La Jolla, CA, USA). The Shapiro–Wilk test was used to confirm whether the data for continuous variables were normally distributed. All data are expressed as mean ± standard deviation (SD). Differences between two groups were assessed using Student's *t*‐test, whereas differences among multiple groups were analyzed using one‐way analysis of variance (ANOVA) followed by Tukey's multiple comparisons test. Statistical significance was set at *p* < 0.05.

## 3 Results

### 3.1 One Week of Feeding With DKT Suppressed Liver IRI

One week before surgery, the mice were divided into two groups: the control group, which received a normal diet (AIN‐93G), and the DKT group, which received a diet supplemented with 3.6% DKT extract.

The amount of food consumed per day and weight gain over 1 week were the same in both groups, confirming that there was no decrease in the amount of food consumed due to the taste of the DKT extract‐mixed diet on day 8 (Supplementary Figure S1).

Ischemia and reperfusion (IR) stimulation was performed after 1 week of preoperative feeding with the assigned diet. At 6 h after reperfusion, the necrotic area was reduced in the DKT group (Figure 1A). Suzuki's histological score was significantly lower in DKT than in CD mice (Figure 1B). To further investigate the extent of cell death 6 h after reperfusion, TUNEL staining was performed (Figure 1C). The number of TUNEL‐positive cells was significantly reduced in DKT mice compared to that in CD mice (Figure 1D). Consistent with the histological findings, liver enzymes were significantly suppressed in the DKT group (Figure 1E).

**FIGURE 1:**
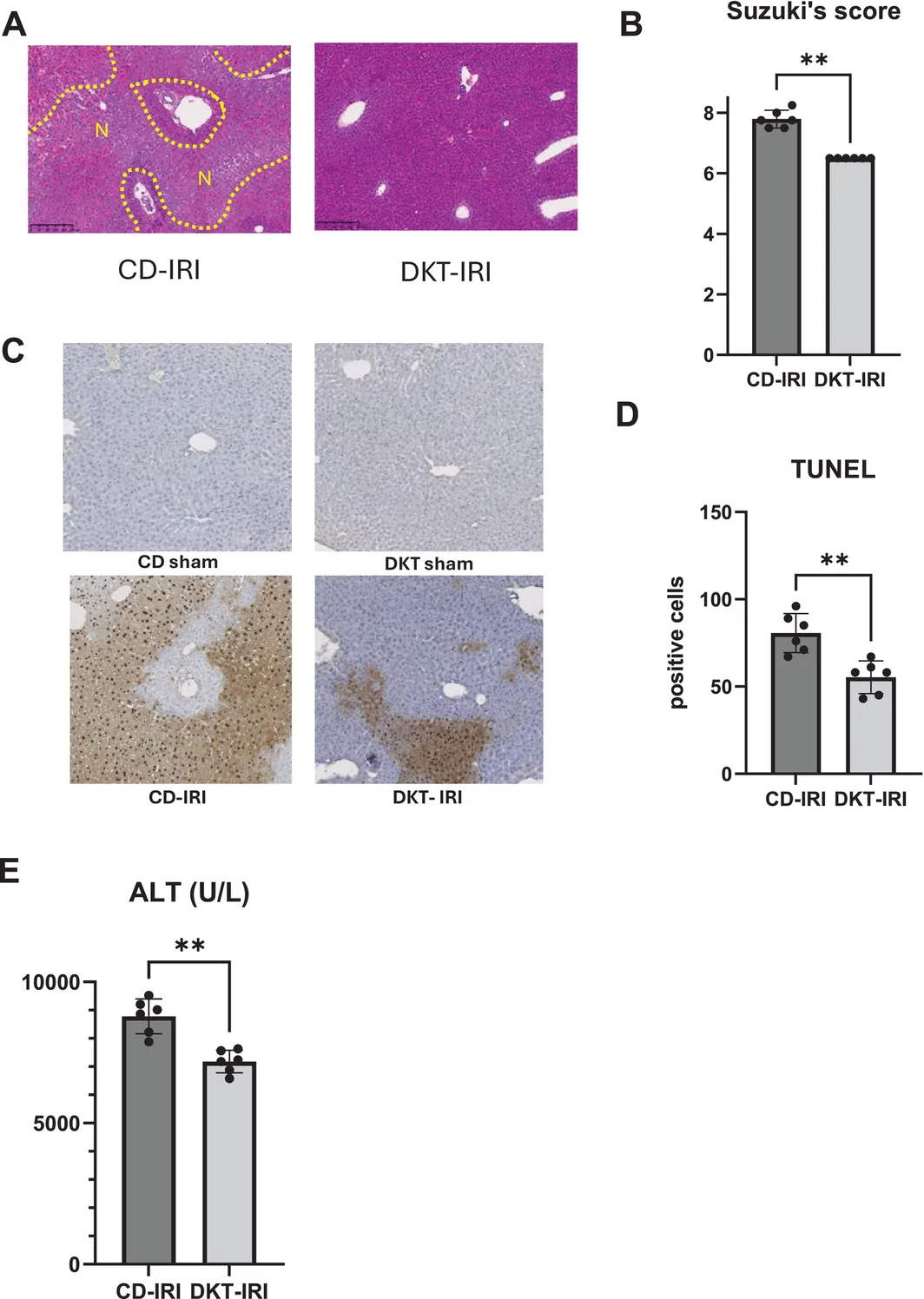
Ischemia and reperfusion (IR)‐induced hepatocellular damage in control diet (CD)‐ and daikenchuto (DKT)‐fed mice. (A) Representative hematoxylin and eosin staining of the liver following IR (magnification, ×400). Yellow dotted lines indicate the necrotic areas (indicated as N). (B) Suzuki's histological score after IR (*n* = 6 mice per group; **p < 0.01). (C) TdT‐mediated dNTP nick end labeling (TUNEL)‐assisted detection of hepatocyte death after IR (magnification, ×400). (D) Quantification of TUNEL‐positive cells (*n* = 6 mice per group; **p < 0.01). (E) Serum alanine aminotransferase (sALT) levels 6 h after reperfusion (*n* = 6 mice per group; **p < 0.01).

To clarify the mechanism of action, we created a single crude drug mixture of the constituent herbal medicines (processed ginger, ginseng, and Japanese zanthoxylum peel) to match the herbal medicine content of the 3.6% DKT extract‐mixed feed and conducted IRI experiments using the same protocol. However, there was no improvement in liver enzyme levels or tissue findings after IR treatment in the groups fed the single crude drug mixture of the three herbal medicines (Figure 2A).

**FIGURE 2:**
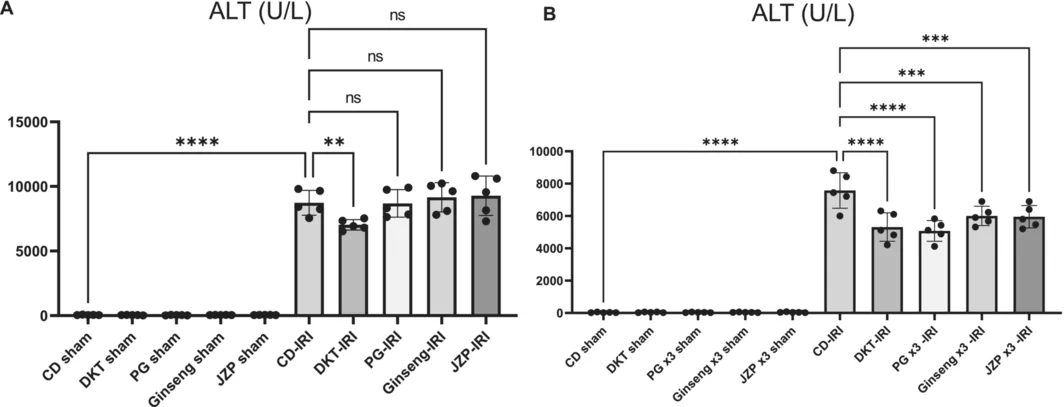
Ischemia and reperfusion (IR)‐induced hepatocellular damage in control diet (CD)‐fed, daikenchuto (DKT)‐fed, and mice fed with each crude drug of DKT. (A) Serum alanine aminotransferase (sALT) levels 6 h after reperfusion (*n* = 5 mice per group; ns, not significant; **p < 0.01, ****p < 0.0001). (B) sALT levels 6 h after reperfusion in mice fed with threefold amount of each component (*n* = 5 mice per group; ns, not significant; ***p < 0.001, ****p < 0.0001). JZP, Japanese zanthoxylum peel; PG, processed ginger.

When the content of the herbal medicine was increased threefold, liver IRI was significantly ameliorated in each gradient group compared to that in the CD group (Figure 2B).

### 3.2 Effects of DKT and Herbal Medicine on Liver IRI

Liver IRI is characterized by excessive production of proinflammatory cytokines, leading to hepatocellular damage [3]. DKT administration in mice led to a significant decrease in the expression levels of proinflammatory cytokines, such as TNF‐α, IL‐1β, and IL‐6, as well as chemokines, such as C‐X‐C motif chemokine ligand (CXCL) 1 and CXCL2, in the liver tissue of DKT‐fed mice (Figure 3A). These findings underscore the anti‐inflammatory effect of DKT. Similarly, administration of three times the amount of processed ginger downregulated inflammatory cytokines, such as TNF‐α, IL‐1β, and IL‐6, as well as chemokines, such as CXCL1 and CXCL2, in the liver, whereas the other two crude drugs showed only partial effects on the downregulation of cytokines and chemokines (Figure 3B).

**FIGURE 3:**
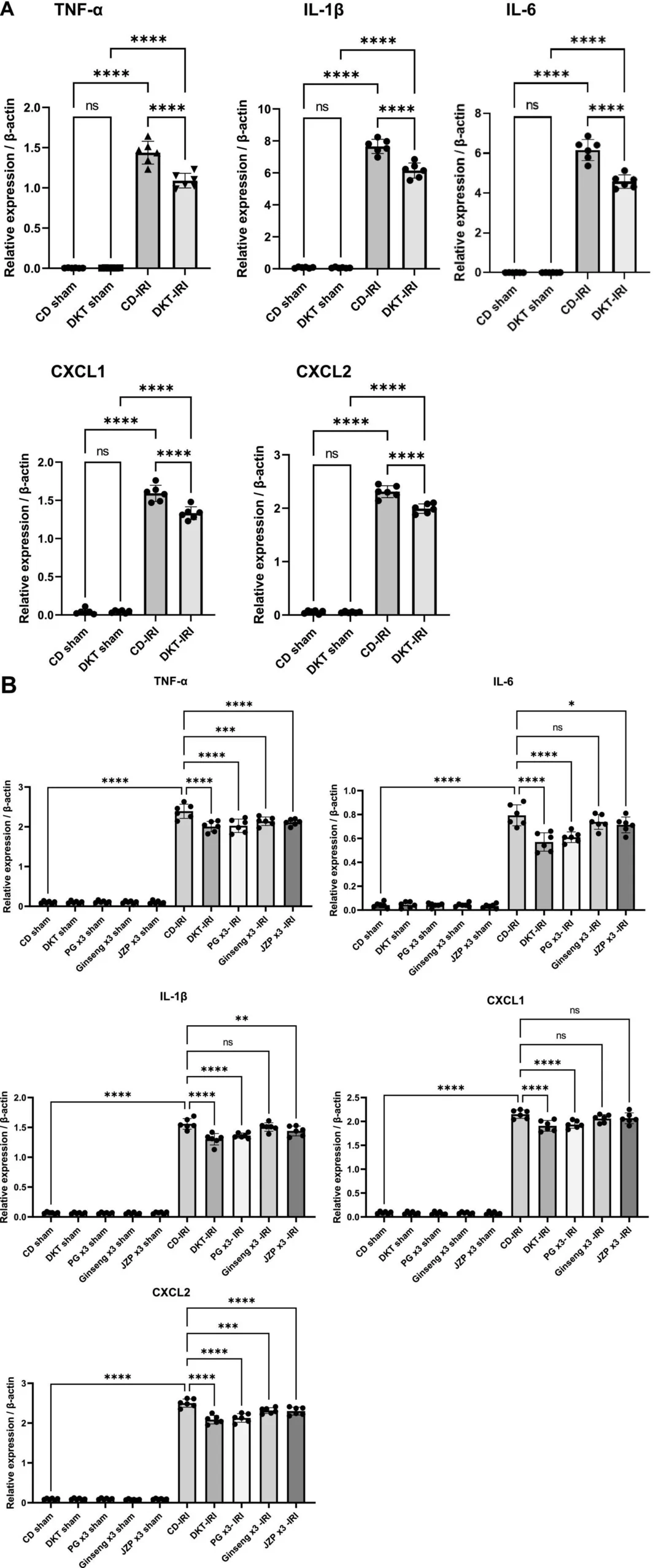
Quantitative real‐time polymerase chain reaction was used to detect proinflammatory cytokines (tumor necrosis factor‐α [TNF‐α], interleukin [IL]‐1β, and IL‐6) and chemokines (C‐X‐C motif chemokine ligand [CXCL] 1 and CXCL2). Data were normalized to the β‐actin gene expression. (A) Expression levels of cytokines and chemokines in control diet (CD) and daikenchuto (DKT)‐fed mice (*n* = 6 mice per group; ns, not significant; ****p < 0.0001). (B) Expression levels of cytokines and chemokines after administration of each crude drug (*n* = 6 mice per group; ns, not significant; *p < 0.05, **p < 0.01, ***p < 0.001, ****p < 0.0001). JZP, Japanese zanthoxylum peel; PG, processed ginger.

### 3.3 HMGB1 Release Was Not Affected by DKT

HMGB1, a DAMP that triggers inflammatory responses [24], activates inflammatory cells through Toll‐like receptor 4 and induces the production of proinflammatory cytokines, thereby contributing to the development of liver IRI [25]. Serum HMGB1 levels increased after IR insult in both CD‐fed and DKT‐fed mice, with no significant difference between the groups (Supplementary Figure 2). These findings suggest that preoperative administration of DKT does not substantially affect HMGB1 release induced by liver IRI.

### 3.4 The Protective Effect of DKT Against Liver IRI Is Not Primarily Mediated by ADM

ADM is frequently reported to be a key mediator of the immunomodulatory effects of DKT [16]. The active ingredients of processed ginger and Japanese zanthoxylum peel stimulate transient receptor potential (TRP) channels in intestinal epithelial cells, leading to the release of ADM into the bloodstream, which exerts anti‐inflammatory effects [15]. Therefore, we hypothesized that preoperative oral administration of DKT stimulates the intestinal tract and increases portal ADM levels, thereby attenuating hepatic IRI.

To test this hypothesis, ADM concentrations in the portal vein were measured after 1 week of feeding with CD or DKT diets. However, no significant differences in portal ADM levels were observed between the CD and DKT groups (Figure 4). Furthermore, portal ADM levels were measured 6 h after reperfusion in the CD and DKT groups. ADM levels were significantly increased by IR stimulation in both CD‐ and DKT‐fed mice, but no significant difference was observed between the two groups (Figure 4). These findings suggest that the protective effect of DKT against hepatic IRI is unlikely to be mediated by DKT‐induced increase in ADM levels.

**FIGURE 4:**
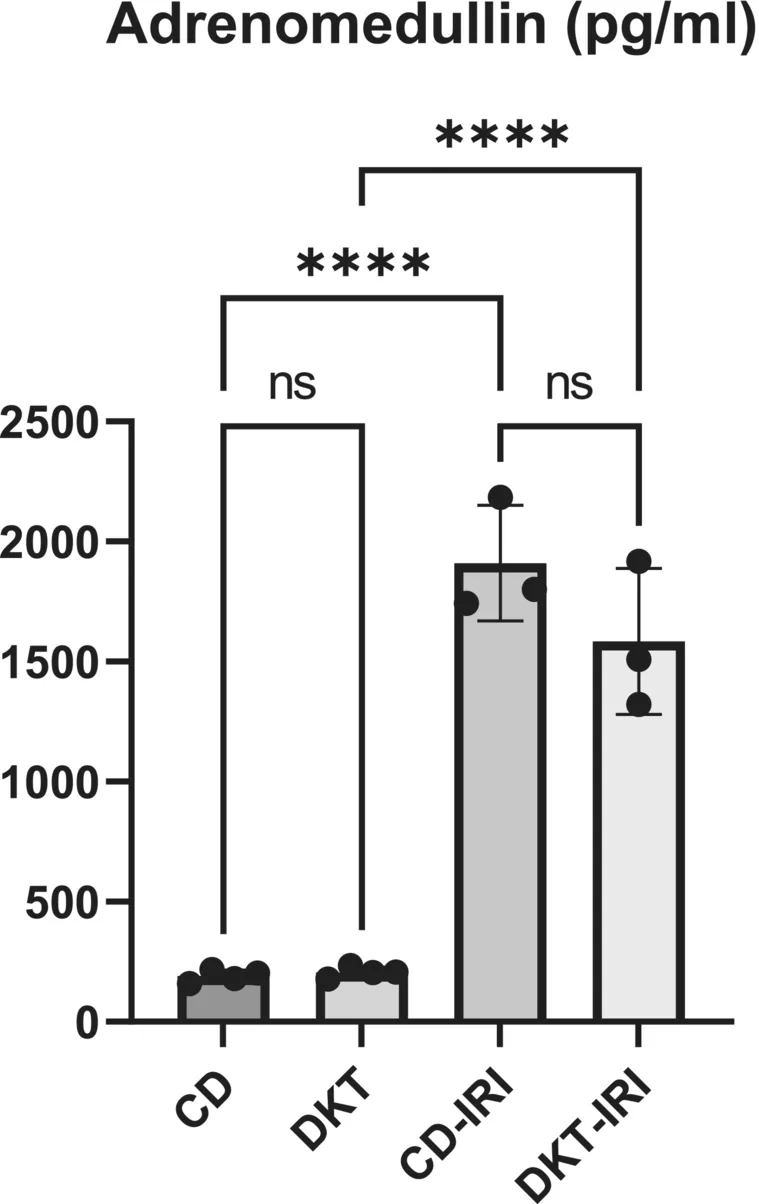
Concentrations of adrenomedullin in the portal vein of control diet (CD)‐sham and daikenchuto (DKT)‐sham mice, as well as CD‐ischemia and reperfusion (IRI) and DKT‐IRI mice at 6 h after reperfusion (sham, *n* = 4 per sham group; *n* = 6 mice per IRI group; ns, not significant; ****p < 0.0001).

### 3.5 DKT and Processed Ginger Increased Expression of Antioxidant Enzyme HO‐1 in Liver Tissues After IR Stimulation

To investigate the mechanism by which DKT attenuates liver IRI, we focused on HO‐1, given its established protective role in liver IRI [26]. We analyzed the expression of HO‐1 in the livers of both CD‐IRI and DKT‐IRI mice and found that HO‐1 expression was significantly upregulated in the DKT‐IRI group (Figure 5A). To further examine the expression of HO‐1 in IR livers, we performed HO‐1 immunostaining of liver tissue and found that HO‐1 expression tended to be higher in non‐parenchymal cells in the DKT‐IRI group (Figure 5B,C). To examine the contribution of HO‐1 to the protective effect of DKT, ZnPP, an HO‐1 inhibitor, was administered intraperitoneally to both CD‐IRI and DKT‐IRI mice. No significant difference in serum ALT levels 6 h after reperfusion was observed between the groups (Supplementary Figure 3), suggesting that HO‐1 may contribute, at least in part, to DKT‐mediated protection against liver IRI.

**FIGURE 5:**
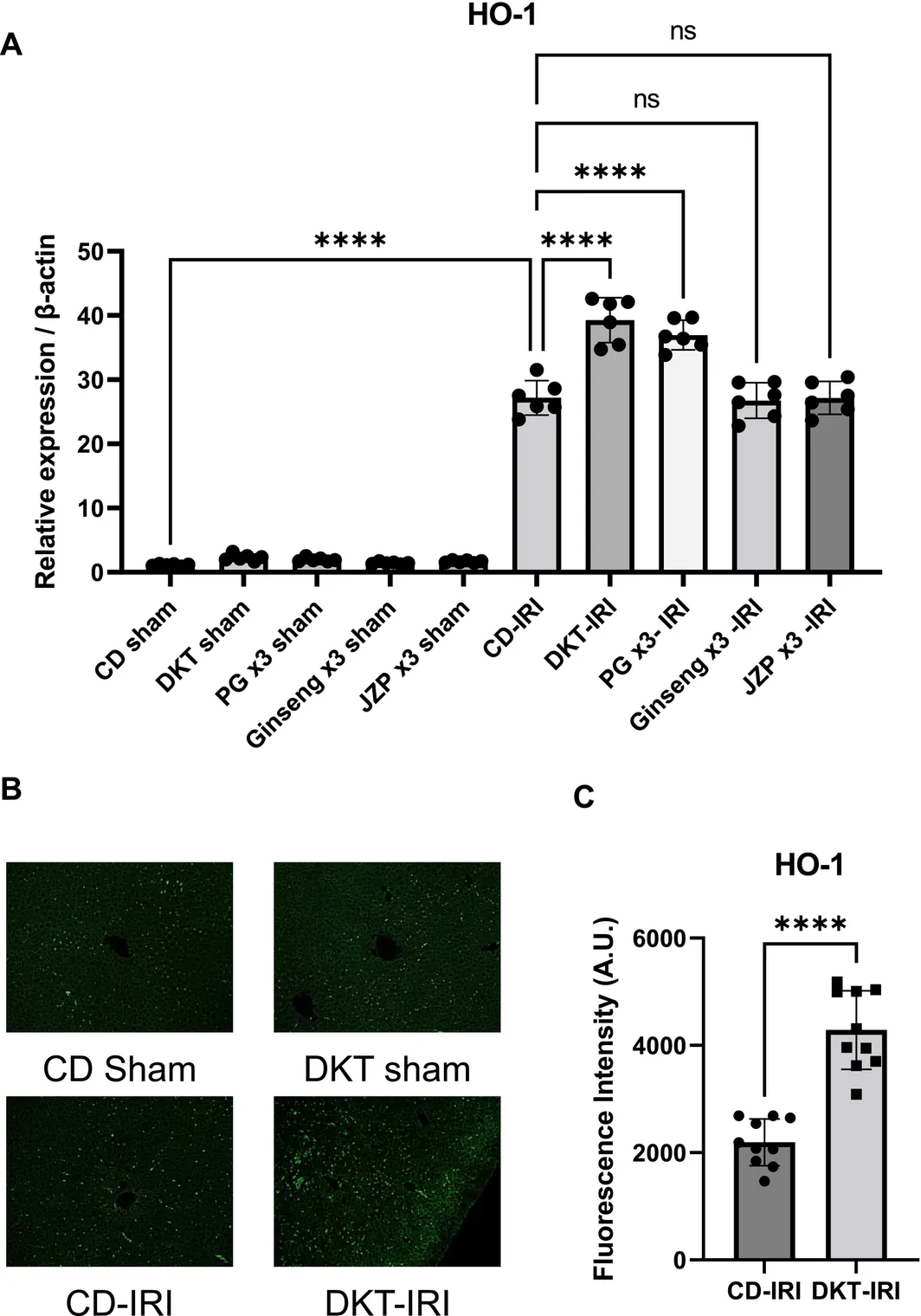
Mechanistic insight into the attenuation of liver ischemia and reperfusion (IRI) in daikenchuto (DKT)‐fed mice. (A) Expression levels of heme oxygenase (HO‐1) in the livers of each group (*n* = 6 mice per group; ns, not significant; ****p < 0.0001). (B) Representative immunofluorescence staining of HO‐1 (green) in control diet (CD) sham, daikenchuto (DKT) sham, CD‐IRI, and DKT‐IRI mice (magnification, ×400). (C) Quantitative analysis of HO‐1 fluorescence intensity. Each dot represents a single cell. Data are shown as mean ± standard deviation (SD) (*n* = 10 cells per group; ****p < 0.0001). AU, arbitrary units; JZP, Japanese zanthoxylum peel; PG, processed ginger.

### 3.6 DKT and Processed Ginger Suppressed Inflammatory Responses in HMGB1‐Activated Macrophage

As macrophage activation is considered a central mechanism underlying hepatic IRI [6], we conducted an intraperitoneal macrophage stimulation experiment to assess the effects of DKT and 6SG on inflammatory responses. Upon stimulation with HMGB1, TNF‐α production was significantly suppressed by both DKT and 6SG treatments in the culture medium (Figure 6). Thus, DKT and 6SG exert protective effects against hepatic IRI by suppressing the proinflammatory activity of macrophages.

**FIGURE 6:**
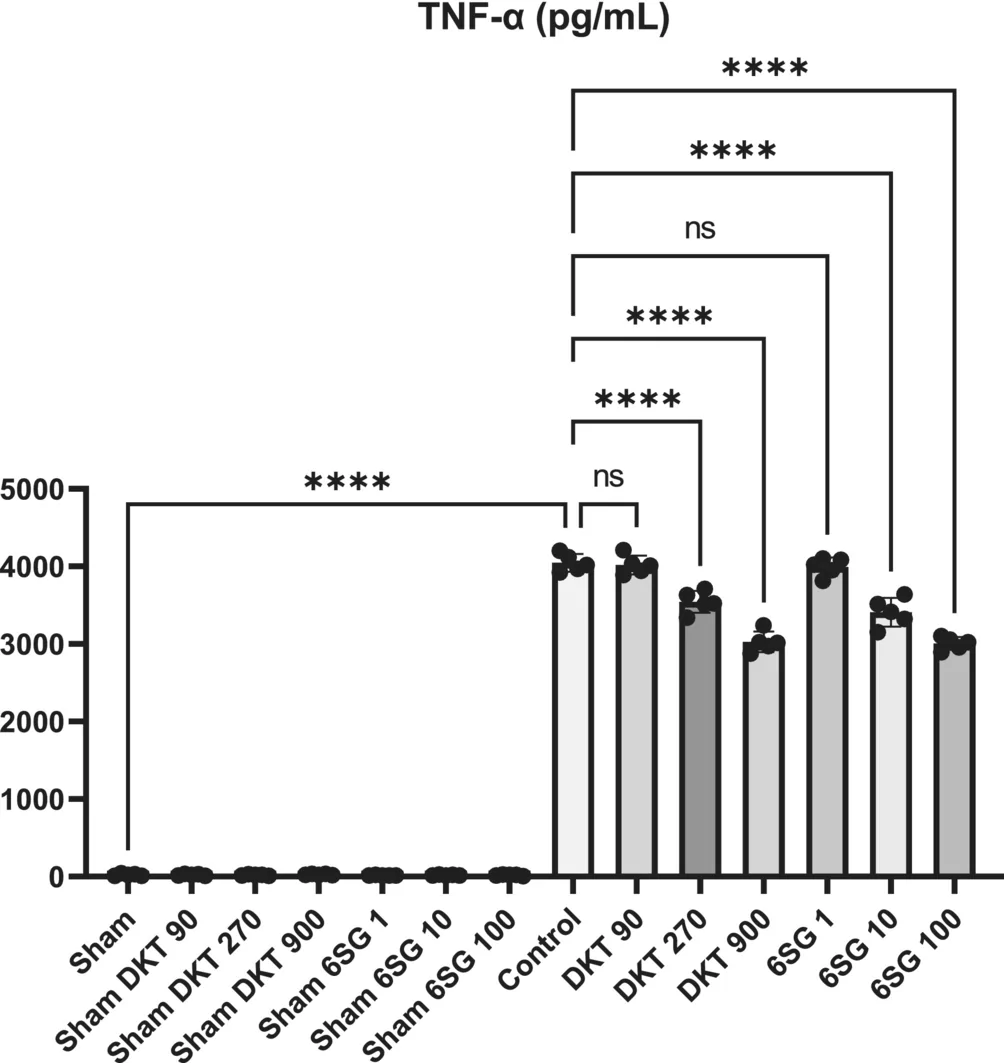
DKT and 6‐shogaol (6SG) suppress the High Mobility Group Box 1 (HMGB1)‐mediated activation of peritoneal macrophages. Peritoneal macrophages from wild‐type (WT) mice stimulated with HMGB1 (1 μg/mL) were cultured with or without pretreatment with DKT or 6SG for 48 h. Tumor necrosis factor‐α (TNF‐α) release was measured by ELISA (*n* = 5 samples per group; ns, not significant; ****p < 0.0001).

## 4 Discussion

Although various strategies have been reported to ameliorate liver IRI, the lack of definitive therapeutic strategies makes liver IRI an unsolved challenge. Interestingly, some Kampo medicines have been reported to have hepatoprotective effects. Inchinkoto has shown possible hepatoprotective effects through the enhancement of antioxidant pathways, such as Nrf2/HO‐1, although clear improvements in major postoperative outcomes have not been demonstrated [27]. In contrast, DKT has been associated with an earlier recovery of bowel function and modest improvement in postoperative gastrointestinal dysmotility after hepatic resection [7, 28, 29]. However, the effects of DKT on IRI remain unclear. In this study, we aimed to confirm whether DKT has beneficial effects on hepatic IRI and to explore its underlying mechanisms of action.

In a previous clinical study, DKT was administered perioperatively at a dose of 15 g/day, starting 3 days before surgery and continuing until Postoperative Day 10 [7]. In the present study, we used a 1‐week preoperative DKT feeding protocol to evaluate the maximum preventive potential of DKT against liver IRI. We found that DKT‐fed mice exhibited less severe liver IRI than CD‐fed mice (Figure 1). To the best of our knowledge, this is the first study to demonstrate that DKT ameliorates liver IRI. DKT has anti‐inflammatory properties that induce ADM release in the intestinal tract [16]. Therefore, we hypothesized that a similar anti‐inflammatory effect might be observed in the liver via increased ADM concentrations in the portal circulation following DKT. However, portal ADM levels did not increase after 1 week of oral DKT administration, and these levels did not differ even after the IR insult between the CD and DKT groups (Figure 4). This suggests that the protective effect of DKT against liver IRI is unlikely to be primarily mediated by increased portal ADM levels.

DKT mainly comprises three crude herbal drugs: processed ginger, ginseng, and Japanese zanthoxylum peel. To investigate the contribution of each crude drug to the protective effect of DKT, we compared liver IRI among mice fed each single crude drug. At doses equivalent to their content in DKT, none of the single crude drugs significantly ameliorated liver IRI (Figure 2A). However, when fed three times the amount of a single crude drug, these mice exhibited significant amelioration of liver IRI compared to CD mice (Figure 2B). These findings suggest that each crude drug possesses hepatoprotective activity, but that the protective effect of DKT cannot be attributed solely to any single constituent at the dose contained in the formulation. Rather, the efficacy of DKT is likely to result from additive or complementary contributions of its constituent crude drugs, as previously reported by Kono et al. [30].

Among the crude drugs, a triple dose of processed ginger alone was the most effective in reducing liver IRI and suppressing the expression of proinflammatory cytokines and chemokines (Figures 2B and 3B), suggesting that processed ginger may be an important contributor to this effect. Nevertheless, because processed ginger at the dose contained in DKT did not significantly ameliorate liver IRI when administered alone, its effect alone does not fully account for the protective effect of the complete DKT formulation. Thus, the effects of ginseng and Japanese zanthoxylum peel, although less pronounced individually, may add to that of processed ginger and contribute to the overall hepatoprotective activity of DKT. Processed ginger has been reported to exhibit anti‐inflammatory effects by suppressing the expression of iNOS and COX‐2 in LPS‐induced liver failure in mice [31].

A previous review summarized that 6SG, a major bioactive compound of processed ginger, can increase cytoprotective HO‐1 expression and that HO‐1 signaling contributes to its anti‐inflammatory and tissue‐protective effects in several experimental models [32, 33]. In the present study, hepatic HO‐1 expression was more prominently induced in DKT‐IRI mice than in CD‐IRI mice (Figure 5A). Previous reports have shown that hepatic HO‐1 expression due to IR insult is predominantly derived from macrophages in both mice and humans [26, 34]. Consistent with these findings, HO‐1‐positive cells in the present study were mainly observed in non‐parenchymal cell areas and exhibited a morphology suggestive of macrophages (Figure 5B).

To further examine the contribution of HO‐1 induction to liver IRI, ZnPP, an HO‐1 inhibitor, was administered to both CD‐fed and DKT‐fed mice 1 h before the ischemic insult, followed by 6 h of reperfusion. As shown in Supplementary Figure 3, serum ALT levels 6 h after reperfusion in ZnPP‐treated DKT‐fed mice (DKT‐IRI ZnPP) were comparable to those in ZnPP‐treated CD‐fed mice (CD‐IRI ZnPP). These findings suggest that HO‐1 induction may contribute, at least in part, to the protective effect of oral DKT against liver IRI. Taken together, DKT, possibly through processed ginger‐derived components, may enhance macrophage‐associated HO‐1 expression, thereby exerting protective effects against liver IR stress.

Furthermore, both DKT and 6SG suppressed the activation of peritoneal macrophages stimulated with HMGB1 in vitro (Figure 6).

These findings indicate that processed ginger and its major active compound, 6‐SG, may contribute to the protective effect of DKT by inhibiting macrophage activation. At the cellular level, both Japanese zanthoxylum peel [35] and ginseng [36] have been reported to exhibit anti‐inflammatory properties. Therefore, the combined administration of these three crude drugs in DKT may provide additive benefits that could account for the observed hepatoprotective effects.

This study has several limitations. First, the absence of differences in portal ADM levels at the examined time points does not completely exclude a transient or local contribution of ADM signaling. Second, component analysis does not fully distinguish true synergistic effects from differences in dose equivalence, extraction efficiency, and bioavailability among the tested formulations [37].

In conclusion, preoperative DKT administration ameliorated hepatic IRI in mice and reduced inflammatory responses in the liver. Processed ginger, particularly its active constituent 6SG, may be a principal contributor to this protective effect. Although the present data do not support a major role for ADM in this setting, they suggest that the modulation of macrophage‐associated inflammatory signaling, potentially accompanied by increased hepatic HO‐1 expression, may underlie the beneficial effects of DKT. Further mechanistic studies are warranted to clarify the synergistic actions of DKT, a formulation composed of three distinct crude drugs in the context of liver IRI.

## Funding

The authors declared that financial support was received for this work and its publication. This study was funded by Tsumura & Co. (Tokyo, Japan), and a Grant‐in‐Aid for Scientific Research B: 20H03743 and a Grant‐in‐Aid for Scientific Research C: 21K08655 from the Ministry of Education, Culture, Science and Sports, Japan.

## Ethics Statement

Animal experimental procedures were approved by the Institutional Animal Welfare and Animal Care Committee of Medical Research Institute KITANO HOSPITAL (approval number: A1900002) and complied with the Guide for the Care and Use of Laboratory Animals (eighth edition).

## Conflicts of Interest

Y.U., H.T., and E.H. received research funding from Tsumura & Co. The other authors declare no conflicts of interest.

## Supporting information


**Supplementary Figure 1.** The body weight of each mouse was monitored on Days 1 and 8 (*n* = 6 mice/group; ns, not significant). BW, body weight; CD, control diet; DKT, daikenchuto.
**Supplementary Figure 2**. Serum HMGB1 levels 6 h after initiation of reperfusion (sham, *n* = 3 per sham group; *n* = 4 mice per IRI group; ns, not significant). CD, control diet; DKT, daikenchuto; HMGB1, High Mobility Group Box 1; IRI, ischemia and reperfusion injury.
**Supplementary Figure 3**. Serum ALT levels 6 h after initiation of reperfusion (sham, *n* = 3 per sham group; *n* = 6 mice per IRI group; *p < 0.05; ns, not significant). CD, control diet; DKT, daikenchuto; IRI, ischemia and reperfusion injury; ZnPP, zinc protoporphyrin IX.

## Data Availability

The data that support the findings of this study are available from the corresponding author upon reasonable request.
