# Supplementary material for: Daikenchuto and Processed Ginger Alleviate Liver Ischemia–Reperfusion Injury by Enhancing Anti‐Inflammatory Effects and HO‐1 Production
Source: JGH Open. 2026 Oct 11;10(10):e70478. doi: 10.1002/jgh3.70478 (PMC13655227; doi:10.1002/jgh3.70478)

Supplementary Figure 1

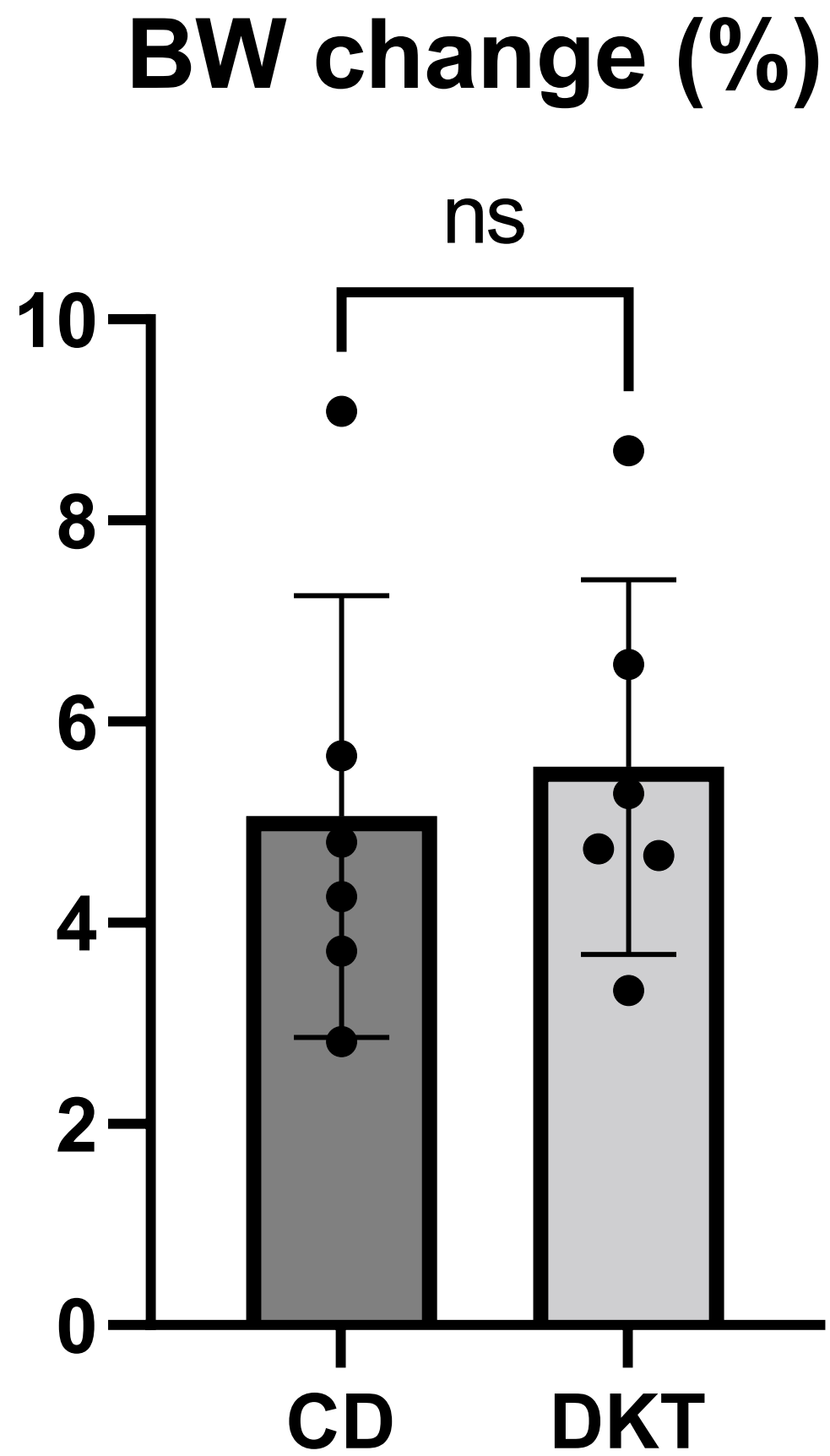

Supplementary Figure 2

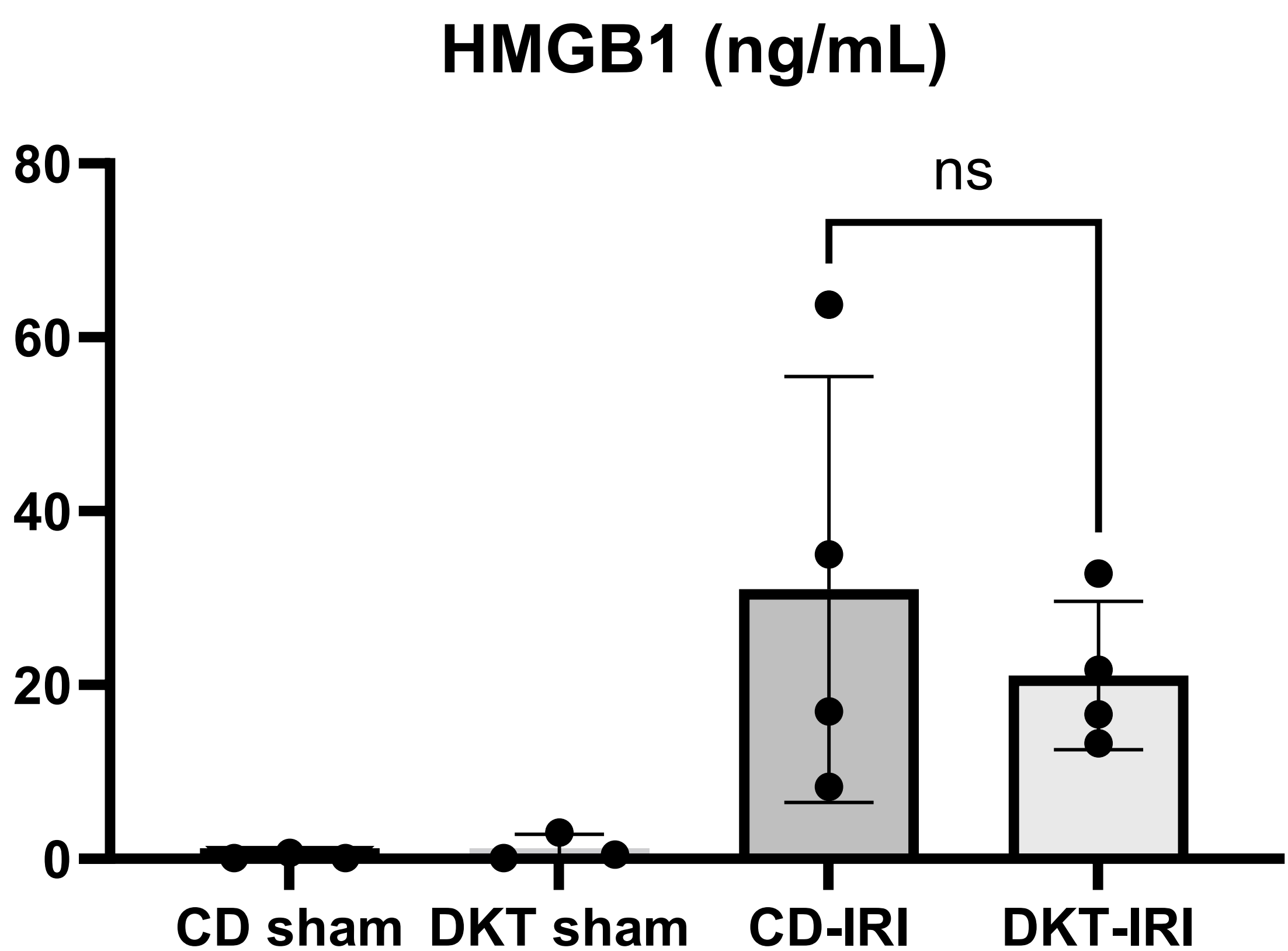

Supplementary Figure 3

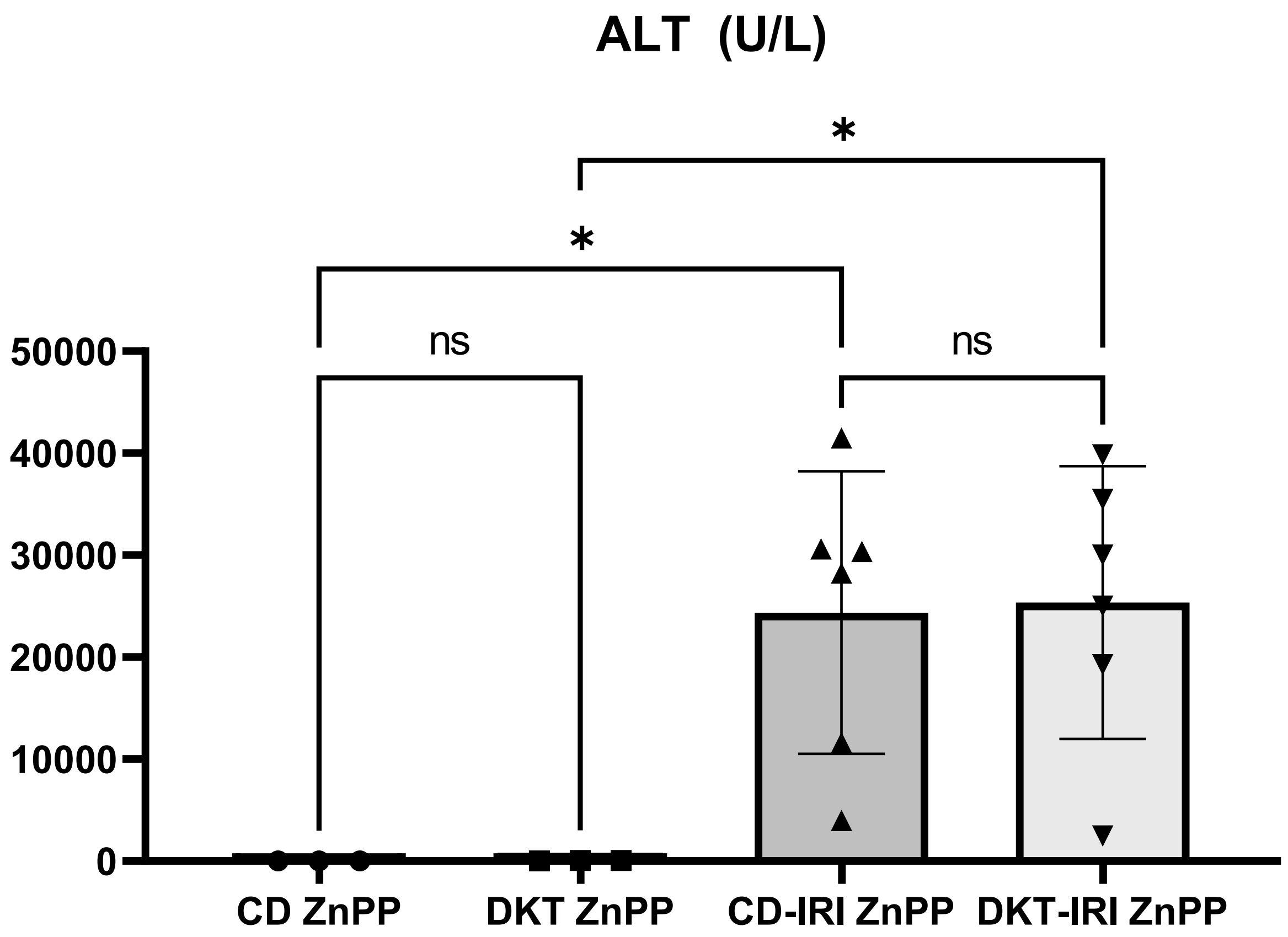

Supplement: Supplementary file 1 [file JGH3-10-e70478-s001.pdf]
